# Clinical management of cancer after kidney transplantation

**DOI:** 10.3389/fimmu.2026.1784083

**Published:** 2026-03-25

**Authors:** Takahisa Hiramitsu, Ayano Ienaga, Yuki Shimamoto, Tomoki Himeno, Yuki Hasegawa, Kenta Futamura, Manabu Okada, Shunji Narumi, Yoshihiko Watarai

**Affiliations:** Department of Transplant and Endocrine Surgery, Japanese Red Cross Aichi Medical Center Nagoya Daini Hospital, Nagoya, Japan

**Keywords:** CAR-T cell therapy, immune checkpoint inhibitors, immunosuppression, kidney transplantation, post-transplant cancer

## Abstract

Advances in immunosuppressive therapy have markedly improved short-term graft survival post-kidney transplantation (KT); however, these advances have also profoundly altered long-term disease patterns in transplant recipients. Cancer remains a major barrier limiting improvements in long-term survival. Kidney transplant recipients are at increased risk of malignancy owing to impaired graft function and immunosuppressive medications. To improve cancer-related outcomes, comprehensive strategies encompassing prevention, early detection through screening, and appropriate treatment are required. Primary prevention includes the appropriate selection and maintenance of immunosuppressive medications, careful consideration of KT indications for recipients and donors with previously treated cancers, and vaccination. Secondary prevention requires effective, risk-adapted cancer screening in high-risk kidney transplant recipients. Following a cancer diagnosis, management includes localized resection and systemic therapy for primary, recurrent, or refractory disease. Systemic therapy includes chemotherapy and immunotherapy, such as immune checkpoint inhibitors and chimeric antigen receptor T-cell therapy, which may affect graft function and cause treatment-specific adverse effects. Additionally, immunosuppressive medications often require modification to minimize the risk of graft rejection while maintaining a low likelihood of recurrence. In this review, we summarize current strategies for cancer prevention, screening, and treatment following KT.

## 1 Introduction

Kidney transplantation (KT) is the optimal treatment for end-stage kidney disease, providing superior survival and quality of life compared with dialysis (1–5). Preservation of long-term graft function is essential to maximize these benefits. To prevent the development of *de novo* donor-specific anti-human leukocyte antigen antibodies and subsequent rejection, immunosuppressive therapy remains indispensable post-KT (6–9). However, long-term immunosuppression creates a unique immunological environment in which cancer has emerged as a major determinant of graft and recipient outcomes (10–13). Consequently, cancer prevention, early detection, and treatment represent major clinical challenges in kidney transplant recipients receiving immunosuppressive medications.

In this review, we summarize current strategies for cancer prevention, screening, and treatment following KT.

## 2 Cancer after kidney transplantation

### 2.1 Epidemiology

The leading causes of recipient mortality and death with a functioning graft (DWFG) post-KT include infection, cardiovascular disease, and cancer. Among these, cancer accounts for approximately 20% of post-transplant deaths and has become a common cause of mortality in kidney transplant recipients (14). Previous studies have shown that cancer-related mortality increases over time post-transplantation (10, 11, 15). Because post-transplant cancer substantially impairs quality of life and long-term survival, it represents a critical clinical issue in KT.

Cancer incidence is significantly higher in kidney transplant recipients than in the general population and in patients undergoing dialysis, largely owing to long-term immunosuppressive therapy (10, 11). Cancer risk is commonly expressed using the standardized incidence ratio (SIR), calculated as the ratio of observed to expected cancer incidence relative to the general population. Overall, the SIR for all cancers among kidney transplant recipients has been reported to be approximately 3.0-fold (SIR: 3.3, 95% confidence interval [CI]: 3.2–3.4) (10).

Cancer incidence post-KT varies markedly according to the cancer type. The most frequent cancer is non-melanoma skin cancer (NMSC) (SIR: 35.9, 95% CI: 33.4–38.5), followed by Hodgkin lymphoma (SIR: 7.59, 95% CI: 6.46–8.92), renal cell carcinoma (SIR: 7.65, 95% CI: 6.41–9.13), melanoma (SIR: 2.97, 95% CI: 2.41–3.66), lung cancer (SIR: 2.88, 95% CI: 2.48–3.34), and colorectal cancer (SIR: 2.16, 95% CI: 1.75–2.66) (10, 11). Conversely, although prostate and breast cancers are the most common malignancies in the general population, their incidence in kidney transplant recipients is comparable to that in the general population, with SIRs of 1.17 (95% CI: 1.00–1.36) and 1.16 (95% CI: 0.93–1.46), respectively (10).

Compared with patients before renal replacement therapy (RRT) or those undergoing dialysis, kidney transplant recipients have a substantially increased risk of cancer (11). The SIRs for patients before RRT and those on dialysis are reported to be 1.16 (95% CI: 1.08–1.25) and 1.35 (95% CI: 1.27–1.45), respectively; the SIR for kidney transplant recipients exceeds 3.0 (SIR 3.27, 95% CI: 3.09–3.46) (11). Cancers known or suspected to be associated with viral infections occur at significantly higher rates after KT than in patients before RRT or those on dialysis. These include cancers associated with human papillomavirus (HPV; cancers of the tongue, oral cavity, vulva, vagina, and penis), Epstein–Barr virus (EBV; Hodgkin disease and non-Hodgkin lymphoma), and hepatitis B or C virus (HBV or HCV; hepatocellular carcinoma) (11).

### 2.2 Impact of cancer on kidney transplantation

Kidney transplant recipients have a higher overall mortality risk than does the general population, regardless of the presence of cancer (16–19). When cancer develops after KT, however, the risk of death increases substantially. The standardized mortality ratio (SMR), which reflects cancer-specific mortality versus the general population, has been reported as approximately 3.0-fold (SMR 2.9, 95% CI: 2.7–3.1) for all cancer types among kidney transplant recipients (15).

The impact of post-transplant cancer on mortality varies considerably by cancer type. Cancer-specific SMRs are particularly high for NMSC (SMR: 51, 95% CI: 43.5–59.6), lymphoma (SMR: 31, 95% CI: 25.6–36.5), and oral and pharyngeal cancers (SMR: 17, 95% CI: 11.0–25.3) (15). In contrast, only modest increases in cancer-related mortality have been reported for multiple myeloma (SMR: 1.5, 95% CI: 0.7–2.9), breast cancer (SMR: 1.1, 95% CI: 0.8–1.6), and prostate cancer (SMR: 1.0, 95% CI: 0.7–1.6) (15). These findings indicate that the prognostic impact of cancer after KT is highly site-specific. Other population-based studies have similarly reported higher cancer-related mortality among kidney transplant recipients than in the general population, with reported SMRs of approximately 2.7 (95% CI: 2.6–2.9) (16). This increased mortality reflects preexisting and *de novo* cancers present during dialysis and developing post-transplantation, respectively (16). The median time from transplantation to cancer-related death has been reported as 3.5 and 8.9 years for recipients with preexisting and *de novo* cancers, respectively (16). Cancer-related SMRs in kidney transplant recipients are comparable with those observed in patients receiving dialysis (SMR: 2.6, 95% CI: 2.5–2.7) (16). However, the spectrum of cancer types associated with the highest mortality differs between these populations. Among patients on dialysis, the highest SMRs are observed for multiple myeloma, testicular cancer, and kidney cancer, whereas among kidney transplant recipients, non-Hodgkin lymphoma, kidney cancer, and melanoma predominate (16).

Several studies have shown that kidney transplant recipients who develop cancer experience significantly worse outcomes than those without cancer (19–21). Approximately 69% of recipients diagnosed with cancer ultimately die from cancer while their graft remains functional (20). Consequently, cancer-related DWFG substantially increases the risk of graft loss in this population (20, 22, 23). The median survival after cancer diagnosis in kidney transplant recipients is approximately 2.1 years, compared with 8.3 years in matched recipients without cancer (20). This markedly shortened survival suggests that cancers in chronic immunosuppression may progress rapidly and lead to early mortality. Cancer-related deaths tend to occur later after transplantation than other fatal complications, such as infection, gastrointestinal disorders, or cardiovascular disease (20). This temporal pattern suggests that although early post-transplant complications receive close clinical attention, malignancies may progress silently and become clinically apparent at more advanced stages. Furthermore, an elevated risk of cancer-related mortality persists even after graft loss (24). Cancer-related SMRs have been reported to be 1.29 (95% CI: 1.21–1.36) in patients receiving dialysis, 2.50 (95% CI: 2.33–2.69) after a first kidney transplant, and 3.00 (95% CI: 2.23–3.96) after a second kidney transplant. In comparison, SMRs among patients returning to dialysis after graft loss were 1.40 (95% CI: 1.00–1.90) and 3.82 (95% CI: 1.75–7.25) after the first and second graft loss, respectively (24). These findings suggest that the oncogenic effects of prior and ongoing immunosuppression may persist beyond graft failure.

Collectively, these data indicate that the development of cancer post-KT is associated with a substantially increased risk of death, strongly influenced by cancer type and stage at diagnosis. Kidney transplant recipients with cancer have significantly poorer graft and patient survival than those without cancer, and DWFG is a major and inevitable contributor to graft loss in this population (20–22).

## 3 Clinical management of cancer

### 3.1 Prevention of cancer

#### 3.1.1 Immunosuppressive medications

Kidney transplant recipients have a substantially higher risk of developing cancer than patients receiving dialysis, a difference largely attributed to long-term exposure to immunosuppressive agents (11–13, 18). Standard maintenance immunosuppressive therapy after KT typically consists of a triple-drug regimen comprising corticosteroids, a calcineurin inhibitor (CNI; cyclosporine or tacrolimus), and either an antimetabolite (most commonly mycophenolate mofetil [MMF]) or a mammalian target of rapamycin (mTOR) inhibitor (everolimus or sirolimus). Here, we discuss preventive strategies for administering these immunosuppressive medications.

##### 3.1.1.1 Induction therapy

In KT, rabbit antithymocyte globulin (ATG), alemtuzumab, and basiliximab are commonly used as induction therapies. The risk of post-transplant lymphoproliferative disorder (PTLD) has been evaluated in kidney transplant recipients, particularly in high-risk settings involving EBV-seropositive donors and EBV-seronegative recipients. In this context, ATG and alemtuzumab were associated with a higher risk of PTLD compared with basiliximab, whereas the risk of PTLD was comparable between ATG and alemtuzumab. These findings suggest that basiliximab may be beneficial in reducing PTLD risk in EBV-seronegative recipients of kidneys from EBV-seropositive donors (25).

However, another study demonstrated that induction with basiliximab was associated with significantly higher rates of acute rejection compared with ATG or alemtuzumab, whereas ATG-based induction was associated with more favorable graft outcomes (26). Taken together, these findings indicate that the selection of induction therapy should be individualized according to recipient EBV serostatus and immunological risk of rejection.

##### 3.1.1.2 Corticosteroids

A systematic review conducted to compare steroid withdrawal or avoidance with steroid maintenance showed that continued corticosteroid use did not increase the risk of cancer within 5 years following KT (risk ratio: 0.77, 95% CI: 0.41–1.46) (27). Similarly, long-term use of low-dose prednisone (5 mg/day) over 10 years was not associated with an increased cancer risk (hazard ratio [HR]: 0.96, 95% CI: 0.64–1.43) (28). These findings suggest that corticosteroids, particularly at low maintenance doses, are unlikely to be a major independent driver of post-transplant cancer.

##### 3.1.1.3 Calcineurin inhibitors

Several studies have shown a dose-dependent association between CNI exposure and cancer risk. In a randomized controlled trial, patients maintained at higher cyclosporine trough levels (150–250 ng/mL) after the first post-transplant year had a significantly higher cancer incidence than did those maintained at lower trough levels (75–125 ng/mL), indicating that excessive CNI exposure increases cancer risk (13). Similarly, higher long-term cumulative exposure to tacrolimus has been associated with an increased cancer risk, particularly in younger recipients. In a retrospective cohort study, higher cumulative tacrolimus exposure was associated with increased cancer risk, with adjusted HRs of 1.57 (95% CI: 1.08–2.28) and 1.31 (95% CI: 1.03–1.66) in 15- and 30-year-old recipients, respectively (28). These findings underscore the importance of closely monitoring and maintaining CNI trough levels at the minimum necessary to prevent *de novo* donor-specific anti-human leukocyte antigen antibodies production and subsequent rejection (6–9). Differences in cancer risk by CNI type have been reported. Tacrolimus has been associated with approximately a two-fold higher incidence of PTLD compared with cyclosporine, regardless of whether it was used in combination with azathioprine or MMF (29). In a large comparative study conducted to evaluate four maintenance regimens (MMF + tacrolimus, MMF + cyclosporine, mTOR inhibitor + tacrolimus, and mTOR inhibitor + cyclosporine), the HR for PTLD was 0.80 (95% CI: 0.65–0.99) in the MMF + cyclosporine group versus the MMF + tacrolimus group (30). When stratified by EBV serostatus, no difference in PTLD risk was observed among EBV-seropositive recipients (HR: 1.00, 95% CI: 0.70–1.42), whereas EBV-seronegative recipients receiving MMF + cyclosporine had a markedly lower risk than those receiving MMF + tacrolimus (HR: 0.45, 95% CI: 0.28–0.72) (30). The combination of an mTOR inhibitor and tacrolimus was associated with an increased risk of PTLD (overall HR: 1.40, 95% CI: 1.03–1.90; HR: 1.98, 95% CI: 1.28–3.07 in EBV-seronegative recipients), suggesting that certain drug combinations may potentiate oncogenic risk rather than mitigate it (30). These studies imply that tacrolimus, or a combination of tacrolimus and an mTOR inhibitor, should be avoided in high-risk PTLD recipients, including EBV-seronegative recipients.

##### 3.1.1.4 Mycophenolate mofetil

Evidence regarding the association between MMF and post-transplant cancer remains inconsistent. A prospective registry-based study using data from the Organ Procurement and Transplantation Network/United Network for Organ Sharing and the Collaborative Transplant Study showed that MMF use was not associated with an increased incidence of PTLD or overall cancer during 3 years of follow-up (31). Conversely, a large retrospective Canadian cohort study reported that higher cumulative MMF exposure over 10 years was associated with an increased risk of all cancers (HR: 1.34, 95% CI: 0.96–1.86) and NMSC (HR: 1.33, 95% CI: 0.98–1.79) (28). Furthermore, long-term MMF exposure was associated with a higher cancer risk in female recipients (HR: 1.86, 95% CI: 1.15–3.00) than in males (HR: 1.16, 95% CI: 0.74–1.81) (28). A recent study conducted to compare MMF trough levels over time before cancer occurrence or graft failure between kidney transplant recipients with and without cancer reported no similar difference between the two groups. These findings suggest that MMF trough levels may not influence cancer development (32). However, MMF trough levels were shown not to be appropriate for monitoring. In previous studies, the calculated area under the MMF level curves was more reliable for monitoring than the trough levels (33, 34). The available data do not provide conclusive evidence that MMF independently increases cancer risk; however, regularly monitoring the calculated area under the MMF level curves and maintaining it within the appropriate range to prevent rejection are necessary.

##### 3.1.1.5 Mammalian target of rapamycin inhibitors

The potential antineoplastic effects of mTOR inhibitors were first suggested in a phase 3 randomized, open-label, multicenter trial conducted to investigate early cyclosporine withdrawal in kidney transplant recipients receiving cyclosporine, sirolimus, and corticosteroids (35). At 3 years post-transplantation, the overall cancer incidence was lower in recipients maintained on sirolimus and corticosteroids than in those continuing cyclosporine-based therapy. Reductions were observed across skin cancer, PTLD, and other cancer categories (35). Furthermore, another randomized trial reported that conversion from CNI to an mTOR inhibitor significantly reduced cancer risk (13). In this study, patients were randomized to continue CNI therapy or to discontinue CNI and switch to CNI-free sirolimus therapy and were followed for 2 years. The overall cancer incidence was significantly lower in the CNI-free group. However, after excluding NMSC, no significant difference between the groups was observed, suggesting that the reduction in overall cancer incidence was largely driven by a decrease in NMSC (36).

Registry data from the Australian and New Zealand Dialysis and Transplant (ANZDATA) Registry further showed that *de novo* use of mTOR inhibitors combined with reduced cyclosporine exposure was associated with significantly lower risks of NMSC (HR: 0.28, 95% CI: 0.11–0.74), non-skin cancer (HR: 0.39, 95% CI: 0.16–0.98), and overall cancer (HR: 0.41, 95% CI: 0.23–0.71), without an increased risk of rejection, graft loss, or mortality (37). Cancer risk appeared to decrease in a dose-dependent manner with increasing mTOR inhibitor exposure, suggesting a potential antitumor effect (37). However, the protective effect of mTOR inhibitors appears to be cancer type-specific. A large registry-based analysis reported that mTOR inhibitor use from the time of transplantation significantly reduced the SIR for NMSC but not for other cancers (38). Further subgroup analysis revealed that this protective effect was limited to basal cell carcinoma, not to squamous cell carcinoma (38). A meta-analysis of individual patient data reported that sirolimus use was associated with a 40% and 56% reduction in overall cancer and NMSC risk, respectively (39). Conversion to sirolimus-based regimens was associated with even greater reductions in cancer incidence (39). However, this benefit was offset by a 1.43-fold increase (95% CI: 1.21–1.71) in overall mortality, indicating that mTOR inhibitor therapy cannot be universally recommended for all kidney transplant recipients (39).

mTOR inhibitors appear to confer a protective effect against NMSC, particularly basal cell carcinoma, whereas their efficacy in preventing other cancers remains uncertain. Moreover, specific drug combinations, such as mTOR inhibitors with tacrolimus, may increase PTLD risk in EBV-seronegative recipients, underscoring the need for individualized risk–benefit assessment (31). Evidence regarding the relationship between blood levels of immunosuppressive medications and cancer is limited; nevertheless, maintaining the lowest effective immunosuppressive dose is recommended to reduce the risk of cancer after KT (13, 28, 32).

#### 3.1.2 Recipient factors

Post-transplant cancers can be broadly classified into infection- and non-infection-related cancers. The risk of infection-related cancer is markedly elevated in kidney transplant recipients, with an SIR of 11.4 (95% CI: 10.7–12.1) compared with that of the general population (10). These cancers include NMSC; cancers of the lip, vulva, vagina, penis, nasal cavity, and sinuses; Hodgkin and non-Hodgkin lymphoma; and cancers of the oral cavity, liver, cervix, and stomach (10). Conversely, non-infection-related cancers show a more modest increase in risk (SIR: 1.97, 95% CI: 1.86–2.09) and include cancers of the kidney, thyroid, lung, gallbladder, pleura, colorectum, small intestine, bladder and urothelium, bone and soft tissue, pancreas, and uterus, as well as malignant melanoma and multiple myeloma (10).

Several oncogenic viral infections are implicated in infection-related post-transplant malignancies, including HBV, HCV, human T-cell lymphotropic virus type 1 (HTLV-1), human herpesvirus 8 (HHV-8), EBV, and HPV (40). Chronic HBV and HCV infections are strongly associated with hepatocellular carcinoma, with reported HRs of 9.84 (95% CI: 4.61–21.0) for HBV, 4.40 (95% CI: 1.85–10.5) for HCV, and 4.63 (95% CI: 1.06–20.2) for HBV/HCV coinfection (41). HTLV-1 infection is thought to increase the risk of adult T-cell leukemia under immunosuppression, although available data remain limited (42, 43). HHV-8 infection is associated with Kaposi sarcoma in immunosuppressed recipients (44). EBV plays a central role in PTLD development, particularly in EBV-seronegative recipients receiving grafts from seropositive donors (45, 46). HPV infection is associated with cervical and vulvar cancers in female recipients, penile cancer in male recipients, and anal cancer in both sexes (47). Vaccination plays a critical role in preventing infection-related cancers. HPV vaccination is recommended for transplant candidates prior to transplantation or during periods of stable graft function, as it reduces the risk of HPV-related malignancies such as cervical and anogenital cancers (47). Vaccination against HBV is also strongly recommended to prevent chronic viral infection and subsequent hepatocellular carcinoma (48).

In kidney transplant recipients with a history of cancer, post-transplant immunosuppressive therapy raises concern regarding the increased risk of cancer recurrence or metastasis. Consequently, an appropriate observation or waiting period pre-transplantation is recommended to ensure that the previously treated cancer is unlikely to recur or progress under post-transplant immunosuppression. Data from the Israel Penn International Transplant Tumor Registry indicate that the overall risk of cancer recurrence after solid organ transplantation (SOT) is approximately 21% (49). Moreover, recipients with a history of cancer pre-transplantation have been shown to have higher all-cause mortality than those without prior cancer, although death is not always attributable to cancer recurrence (50–52). In a population-based cohort study, recipients with pretransplant cancer had an increased risk of cancer-specific mortality (HR: 1.85, 95% CI: 1.20–2.86) and non-cancer-related death (HR: 1.29, 95% CI: 1.08–1.54) compared with those of recipients without a history of cancer (50). Other studies have similarly reported inferior survival outcomes in transplant recipients with a prior cancer. In one large registry analysis, the risk of all-cause mortality after SOT was 30% higher in recipients with a pretransplant cancer history than in those without such a history (HR: 1.30, 95% CI: 1.10–1.50) (51).

The risks of cancer recurrence and mortality were closely associated with the intrinsic recurrence risk of the original cancer (50). These findings highlight the need for careful patient selection and individualized risk assessment when considering KT in candidates with a history of cancer. The optimal duration of the waiting period between cancer treatment and KT varies by cancer type, stage, and response to therapy. Recently, consensus expert opinion statements have been published to guide clinical decision-making by providing recommended waiting times before KT based on cancer type and disease stage at the time of treatment (**Table 1**) (53–58). These recommendations emphasize balancing the risks of cancer recurrence against the well-established survival and quality-of-life benefits of KT.

**Table 1:** Waiting period for the treated cancers of the kidney transplant candidates mentioned in the consensus expert opinion statements.

Consensus expert opinion statements		AST^52^	AST^53^	ITSCC^51^	KDIGO^56^
Cancer type	Non-melanoma skin cancer			○	
	Melanoma		○	○	○
	Thyroid cancer				○
	Breast cancer	○			○
	Lung cancer	○			○
	Colon and rectum cancer	○			○
	Renal cancer	○			○
	Prostate cancer	○			
	Gynecologic cancer	○			○
	Testicular cancer				○
	Bladder cancer	○			○
	Hematologic cancer		○		○

AST, The American Society of Transplantation; ITSCC, International Transplant Skin Cancer Collaborative; KDIGO, Kidney Disease Improving Global Outcomes. This table was cited from the article written by Hiramitsu et al. (55).

#### 3.1.3 Donor factors

Owing to donor shortages, the use of older donors has increased in living and cadaveric donor KT (59, 60). A previous study reported that the risk of cancer transmission is significantly associated with donor age ≥45 years (odds ratio: 9.0, 95% CI: 1.20–69.6) (61). Donor-related cancers are classified as donor-transmitted cancer (DTC) or donor-derived cancer (DDC). DTC refers to a malignancy that is present or presumed to be present in the graft at the time of transplantation (62), whereas DDC refers to a malignancy that was not detectable at the time of transplantation but subsequently develops within the graft following transplantation (62).

In a report from the United Kingdom Transplant Registry and a database search across transplant centers, donor-related cancers were identified in 18 of 30765 recipients (0.06%). Among these, DDC and DTC were identified in three (0.01%) and 15 (0.05%) recipients, respectively (61). DDC transmission can cause substantial morbidity and mortality in kidney transplant recipients (61, 62). A systematic review reported outcomes for 234 recipients with DTC after KT. The most common DTCs were lymphoma (20.5%), renal cancer (17.9%), melanoma (17.1%), and non-small cell lung cancer (5.6%) (62). The poorest prognoses were observed in melanoma and lung cancer (5-year overall survival, 43% and 19%, respectively), whereas renal cell cancer and lymphoma (5-year overall survival, 93% and 63%, respectively) were associated with more favorable prognoses (62). Additionally, DTC diagnosed within 6 weeks post-transplantation was associated with better outcomes than DTC diagnosed later (61).

In living donor KT, donors undergo meticulous evaluation to rule out active malignancy or recurrence of previously treated cancer. Conversely, in cadaveric donor KT, ruling out active or previously treated recurrent malignancy is more challenging because of the limited time and modality for cancer diagnosis before donation. In a previous report, donor-related cancers were not identified in living donor transplants, but were identified in 0.14% of brain-dead donors and 0.24% of donors after cardiac death among 14,986 donors (61). To reduce the risk of cancer transmission in living and cadaveric donor transplantation, guidelines have been published to guide donor eligibility in individuals with a prior history of cancer (63–66). By adhering to these guidelines, the risk of donor-related cancers can be minimized.

#### 3.1.4 Environmental factor

Environmental factors also contribute to cancer risk post-KT. Ultraviolet radiation exposure can cause DNA damage and increase the risk of skin cancer (67). Immunosuppressive medications further amplify the risk of ultraviolet radiation-related skin cancers in kidney transplant recipients (68, 69). Cumulative sunlight exposure has been identified as an independent risk factor for NMSC after KT (70). Accordingly, sun-protective strategies should be taken to prevent skin cancer (71).

### 3.2 Cancer screening

In the general population, cancer screening facilitates early detection and treatment of cancer, reducing cancer-related mortality and healthcare costs (72). Given the substantially increased risk of cancer development and cancer-related death among kidney transplant recipients, largely driven by long-term immunosuppressive therapy, the importance of cancer screening is even greater in this population. The Kidney Disease: Improving Global Outcomes clinical practice guidelines recognize the importance of cancer screening in kidney transplant recipients (40). To date, various review articles and guidelines have proposed screening strategies tailored to SOT recipients (40, 73–80).

A recent study from our center reported that cancer screening was associated with reduced DWFG (32). In that study, DWFG occurred more frequently in recipients with cancer than in those without cancer (HR: 5.632, 95% CI: 3.252–9.756). Additionally, kidney transplant recipients with cancer detected incidentally or symptomatically had significantly worse DWFG-free survival than recipients without cancer (HR: 8.337, 95% CI: 4.372–15.897) and those whose cancer was detected through screening (HR: 2.088, 95% CI: 0.983–4.436) (32). Routine surveillance in this study included regular blood testing, chest radiography, fecal occult blood testing, and computed tomography. Transplant recipients were also encouraged to undergo standard age- and sex-appropriate cancer screening as part of routine health maintenance, including upper gastrointestinal endoscopy, breast cancer screening, and cervical cancer screening (32). Although the cost-effectiveness of screening was not evaluated, given the elevated incidence of cancer and the poorer prognosis associated with post-transplant malignancy, most experts support risk-adapted screening approaches in high-risk populations.

In addition to immunosuppressive therapy, multiple recipient- and disease-related factors contribute to cancer risk post-KT. Reported risk factors include longer duration of dialysis, male sex, smoking, a history of cancer, re-transplantation, older age, higher body mass index, exposure to immunosuppressive agents pre-transplantation, and oncogenic viral infections (10, 20, 81–87). A large Nordic population-based study involving 12,984 kidney transplant recipients revealed that male sex, increasing age at transplantation, and a history of cancer before transplantation were independent risk factors for post-transplant cancer (10). Female recipients had a 27% lower cancer risk than did male recipients (HR: 0.73, 95% CI: 0.66–0.81). In contrast, dialysis (HR: 1.01, 95% CI: 0.83–1.23), donor type (living vs. deceased; HR: 0.95, 95% CI: 0.84–1.06), and underlying kidney disease (HR: 0.87–1.17) were not associated with an increased cancer risk (10). However, a history of cancer pre-transplantation increased the risk of post-transplant cancer by 36% (HR: 1.36, 95% CI: 1.14–1.62) (10). Cancer risk also increased with advancing age at first transplantation, with HRs of 0.33 (95% CI: 0.29–0.37) for recipients aged <50 years, 1.77 (95% CI: 1.58–1.99) for those aged 60–69 years, and 2.42 (95% CI: 2.01–2.91) for those aged ≥70 years, compared with recipients aged 50–59 years (10).

Other studies have suggested possible associations between certain primary kidney diseases and post-transplant cancer risk. In some cohorts, autosomal dominant polycystic kidney disease (HR: 1.262, 95% CI: 1.065–1.489) and diabetic nephropathy (HR: 1.506, 95% CI: 1.145–1.963) were reported to be associated with a modestly increased risk, although these findings have not been consistently observed across studies (81). Furthermore, exposure to immunosuppressive therapy before transplantation for treating glomerulonephritis has been associated with an increased risk of post-transplant cancer (HR: 1.82, 95% CI: 1.10–3.00) (82). In that study, pretransplant exposure to cyclophosphamide (HR: 2.59, 95% CI: 1.48–4.55), rituximab (HR: 3.82, 95% CI: 1.69–8.65), or both (HR: 4.44, 95% CI: 1.88–10.84) significantly increased cancer risk, whereas pretransplant use of CNIs (HR: 0.96, 95% CI: 0.29–3.21) or MMF (HR: 1.73, 95% CI: 0.83–3.60) did not (82). Data from the ANZDATA Registry indicate that among kidney transplant recipients with a history of primary cancer, recurrent (0.8%) and second primary (2.0%) cancers were relatively uncommon, whereas most recipients (97.2%) developed a first post-transplant cancer (88).

Particular attention should be paid to recipients at high risk for infection-related cancers. As mentioned in the prevention section, some infection-related cancers can be prevented through vaccination. However, cancers associated with HHV8, HTLV-1, and EBV cannot be prevented via vaccination at present. Additionally, treated HCV and HBV are still risk factors for developing hepatocellular cancer (89–91). Kidney transplant recipients with existing HPV infection are at high risk of developing HPV-related cancers, although a recent study has shown the preventive effect of the HPV vaccine on recurrence of the treated high-grade cervical intraepithelial neoplasia in the general population (92).

Robust evidence showing the effectiveness or cost-effectiveness of cancer screening in kidney transplant recipients remains lacking; however, screening kidney transplant recipients with the previously mentioned high-risk factors for cancer may effectively detect cancers at an early stage and could improve the graft and recipient survival.

### 3.3 Cancer treatment

The treatment for the cancer after KT includes operation, chemotherapy, immune checkpoint inhibitors (ICPIs), and chimeric antigen receptor (CAR) T-cell therapy. Both immunosuppressive medications and these cancer treatments can affect operative outcomes, graft function, and graft survival.

#### 3.3.1 Surgery

Surgical resection remains a cornerstone of cancer treatment in kidney transplant recipients, particularly for localized and potentially curable cancers. Compared with systemic therapies such as chemotherapy or ICPIs, surgery offers a definitive oncologic approach, making it a preferred first-line treatment when technically feasible.

A key consideration in cancer surgery post-KT is perioperative immunosuppressive management. Complete withdrawal of immunosuppression is generally avoided owing to the high risk of acute allograft rejection. Clinically, CNIs are typically continued with careful therapeutic drug monitoring, whereas antimetabolites such as MMF or azathioprine may be temporarily reduced or withheld to lower the risk of infection and impaired wound healing (93). Corticosteroids are usually maintained, with stress-dose supplementation considered for major surgical procedures. mTOR inhibitors require particular attention, as they are associated with delayed wound healing and may require discontinuation during the perioperative period (93). Additionally, concomitant use of an mTOR inhibitor and MMF can further increase the risk of delayed wound healing (93, 94).

#### 3.3.2 Chemotherapy

Chemotherapy remains an important treatment modality for advanced or unresectable cancers in kidney transplant recipients. However, its use in this population requires careful consideration because of altered pharmacokinetics, drug–drug interactions between chemotherapeutic agents and immunosuppressive medications, baseline renal dysfunction, chronic immunosuppression, and the risk of allograft injury (95).

A major challenge in administering chemotherapy after KT is preserving allograft function. Many cytotoxic agents, including platinum-based compounds, methotrexate, gemcitabine, and ifosfamide, are partially or predominantly renally excreted and may cause direct nephrotoxicity (95, 96) through acute tubular toxicity, acute tubulointerstitial nephritis, or glomerular injuries (97–99). In addition to previously mentioned conventional chemotherapies, targeted cancer agents such as anti-vascular endothelial growth factor agents, tyrosine kinase inhibitors, B-Raf proto-oncogene inhibitors, anaplastic lymphoma kinase inhibitors, endothelial growth factor receptor inhibitors, Bcr-abl tyrosine kinase inhibitors, and rituximab have also been associated with drug-induced acute kidney injury (AKI) (96). Reduced renal reserve in transplant recipients increases susceptibility to AKI, which can precipitate chronic allograft dysfunction or graft loss. Therefore, dose adjustment based on estimated glomerular filtration rate and close monitoring of renal function are essential during chemotherapy administration (95).

Drug–drug interactions between chemotherapeutic agents and immunosuppressive medications represent another critical concern. CNIs and mTOR inhibitors are metabolized via cytochrome P450 pathways, which may be inhibited or induced by certain anticancer drugs, leading to unpredictable immunosuppressant exposure. Such interactions may result in under-immunosuppression with an increased risk of rejection or over-immunosuppression with heightened toxicity and infection risk. Frequent therapeutic drug monitoring is therefore strongly recommended (95).

Myelosuppression is often more pronounced in kidney transplant recipients receiving chemotherapy. Chronic immunosuppression, impaired bone marrow reserve, and prior viral infections increase the risk of severe neutropenia, anemia, and thrombocytopenia (95, 100, 101). Consequently, transplant recipients are more susceptible to life-threatening infections and treatment interruptions (100). Prophylactic use of recombinant granulocyte colony-stimulating factor and early intervention for febrile neutropenia are recommended (100, 102, 103). Infection risk is further amplified by the combined effects of chemotherapy-induced neutropenia and baseline immunosuppression (100, 102), with reports of increased opportunistic infections, including cytomegalovirus reactivation and invasive fungal infections (100, 102).

Immunosuppressive therapy adjustment during chemotherapy must be individualized. In many cases, antimetabolites, such as MMF, are reduced or temporarily discontinued to mitigate overlapping myelotoxicity, whereas CNIs are maintained at the lowest effective dose to prevent rejection (95, 101). Complete withdrawal of immunosuppression is generally avoided because of the high risk of acute rejection. Overall, chemotherapy in kidney transplant recipients requires a tailored approach that balances oncologic efficacy with graft preservation and infection prevention.

#### 3.3.3 Immune checkpoint inhibitors

ICPIs have shown substantial efficacy in treating various advanced malignancies in the general population. These agents include inhibitors targeting programmed cell death 1, programmed cell death ligand 1, and cytotoxic T-lymphocyte-associated antigen 4.

Experience with ICPI therapy in kidney transplant recipients remains limited; however, accumulating evidence indicates that ICPIs can achieve meaningful antitumor responses in this population. Simultaneously, ICPI use is associated with a high risk of allograft rejection. In a multicenter study of kidney transplant recipients treated with ICPIs, approximately 42% of patients developed acute rejection, typically within 2 months of treatment initiation, and 65% of those experiencing rejection ultimately lost graft function (104). When graft rejection occurs after ICPI administration, therapy is typically discontinued, and steroid pulse or T-cell–depleting therapies are administered, as most rejections are T-cell–mediated vascular rejections rather than antibody-mediated (95). A recent meta-analysis reported that ICPI withdrawal owing to acute rejection occurred in over 40% of cases (105).

Strategies to mitigate rejection risk during ICPI therapy have been proposed but remain inadequately studied. The use of mTOR inhibitors and maintenance triple-drug immunosuppressive regimens appears to be associated with lower rejection rates, although these findings are based on observational findings rather than randomized comparisons (104). Currently, no evidence-based guidelines exist regarding optimal immunosuppressive management before or during ICPI therapy in kidney transplant recipients. Proposed approaches include initiating corticosteroids concomitantly with ICPI therapy, followed by tapering to a maintenance dose, or converting to an mTOR inhibitor-based immunosuppressive regimen before ICPI initiation (106). However, these strategies are based primarily on expert opinion and small case series. Further prospective studies are needed to establish safe and effective protocols that balance oncologic efficacy with graft preservation in kidney transplant recipients receiving ICPI therapy.

ICPIs represent a major advance in cancer therapy; however, their application in kidney transplant recipients is associated with a substantial risk of acute rejection. Further mechanistic studies and carefully designed prospective trials are needed to identify strategies that can safely balance antitumor immunity with graft tolerance in this unique population.

#### 3.3.4 Chimeric antigen receptor T-cell therapy

CAR T-cell therapy has emerged as a highly effective treatment for relapsed or refractory B-cell cancers, including recurrent and refractory diffuse large B-cell lymphoma. By genetically engineering autologous T cells to express tumor-specific antigen receptors, CAR T-cell therapy induces potent and sustained antitumor immune responses (107). However, its application in kidney transplant recipients remains highly challenging and largely experimental because concurrent use of immunosuppressive medications is typically prohibited in CAR T-cell therapy trials (108). A major concern is the risk of acute graft rejection. Additionally, cytokine release syndrome and immune effector cell–associated neurotoxicity syndrome are common and potentially life-threatening complications.

A recent multicenter retrospective analysis conducted to evaluate the safety and efficacy of CAR T-cell therapy for adults with relapsed or refractory SOT-associated PTLD involved 22 recipients, 14 of whom were kidney transplant recipients, and reported that immunosuppressive medications were completely discontinued before CAR T-cell infusion in 14 (64%). Among these, immunosuppressive therapy was reintroduced in 11 recipients 3 months after CAR T-cell therapy. Graft rejection occurred in three kidney transplant recipients (14%). Cytokine release syndrome and immune effector cell–associated neurotoxicity syndrome occurred in 18 (82%) and 16 recipients (72%), respectively. However, the overall response rate to CAR T-cell therapy was 64%, with 55% complete response, 9% partial response, 18% stable disease, and 18% progressive disease (109).

These findings indicate that while CAR T-cell therapy represents a revolutionary advance in cancer immunotherapy, its role in kidney transplant recipients remains undefined. Careful patient selection, individualized immunosuppressive strategies, and close monitoring of graft function are essential, and further studies are needed to establish safety and efficacy in this unique population.

#### 3.3.5 Modification of immunosuppressive agents

The optimal strategy for immunosuppressive management after cancer diagnosis and treatment in kidney transplant recipients remains unclear. As immunosuppressive therapy contributes to tumor progression, recurrence, and metastasis, clinicians frequently consider dose reduction or modification of immunosuppressive regimens in clinical practice. However, there is currently no consensus on which agents should be reduced or discontinued, or on the extent to which immunosuppression can be safely minimized (40, 56). Conversion to an mTOR inhibitor-based regimen is often considered because of the potential antitumor effects of these agents. The European Society for Organ Transplantation Transplant Learning Journey Project addressed two key clinical questions (1): whether immunosuppression should be reduced after cancer diagnosis and (2) whether conversion to an mTOR inhibitor-based regimen should be considered (110). Regarding dose reduction, lowering CNI exposure may theoretically reduce the risk of cancer recurrence or metastasis. However, this approach must be balanced against the risk of graft rejection. In a randomized study of low-immunological-risk, steroid-free kidney transplant recipients, a 50% reduction in extended-release tacrolimus dosing, targeting trough levels >3 μg/L, was associated with significantly higher rates of acute rejection and *de novo* donor-specific antibody formation than was standard dosing (target trough levels: 7–12 μg/L) (111). These findings underscore the potential hazards of abrupt or excessive CNI dose reduction.

Alternatively, conversion from CNI-based therapy to an mTOR inhibitor has been shown to reduce the recurrence risk of NMSC by approximately 50% (112). However, such conversion has been associated with increased risks of mortality, graft failure, and chronic rejection in some studies, indicating that this strategy should be reserved for carefully selected patients (110). Switching from a CNI plus MMF regimen to a CNI plus mTOR inhibitor regimen may represent a more balanced approach. Robust evidence showing reduced cancer recurrence with this strategy remains lacking; however, available data suggest that combination therapy carries a lower risk of mortality, graft loss, and acute rejection than complete replacement of CNIs with mTOR inhibitors (110).

Currently, no standardized protocol exists for immunosuppressive management after cancer treatment in kidney transplant recipients.

## 4 Conclusions

Kidney transplant recipients face a substantially increased risk of cancer as a consequence of long-term immunosuppressive therapy, and the development of cancer has a profound adverse impact on graft survival and patient outcomes. Cancer has emerged as a leading cause of death after KT, particularly in the long-term post-transplant period. The risk of post-transplant cancer is influenced by multiple factors, including the intensity and type of immunosuppressive therapy, recipient characteristics, prior cancer history, and oncogenic viral infections. Certain immunosuppressive strategies, such as minimizing CNI exposure or incorporating mTOR inhibitors, may reduce the incidence of specific cancer types; however, these approaches must be carefully balanced against the risks of rejection, graft loss, and mortality. Management of immunosuppression after cancer diagnosis remains challenging because high-quality evidence to guide clinical decision-making is limited. Emerging cancer therapies, including ICPIs and CAR T-cell therapy, offer new treatment options but pose substantial risks to graft survival. Given these complexities, individualized risk assessment, cancer prevention, vigilant long-term follow-up, and implementation of risk-adapted cancer screening strategies are essential to improving long-term outcomes in kidney transplant recipients. Further prospective studies are needed to establish evidence-based approaches for cancer prevention, screening, immunosuppressive management, and treatment in this vulnerable population.

## Glossary

AKI: acute kidney injury
ANZDATA: Australian and New Zealand Dialysis and Transplant Registry
ATG: antithymocyte globulin
CAR: chimeric antigen receptor
CI: confidence interval
CNI: calcineurin inhibitor
DDC: donor-derived cancer
DTC: donor-transmitted cancer
DWFG: death with a functioning graft
EBV: Epstein–Barr virus
HBV: hepatitis B virus
HCV: hepatitis C virus
HHV-8: human herpesvirus 8
HPV: human papillomavirus
HR: hazard ratio
HTLV-1: human T-cell lymphotropic virus type 1
ICPI: immune checkpoint inhibitor
KT: kidney transplantation
MMF: mycophenolate mofetil
mTOR: mammalian target of rapamycin
NMSC: non-melanoma skin cancer
PTLD: post-transplant lymphoproliferative disorder
RRT: renal replacement therapy
SIR: standardized incidence ratio
SMR: standardized mortality ratio
SOT: solid organ transplantation.

## References

[1] Tonelli M Wiebe N Knoll G Bello A Browne S Jadhav D . Systematic review: kidney transplantation compared with dialysis in clinically relevant outcomes. Am J Transplant. (2011) 11:2093–109. doi: 10.1111/j.1600-6143.2011.03686.x, PMID: 21883901

[2] Rose C Gill JS Gill JS . Association of kidney transplantation with survival in patients with long dialysis exposure. Clin J Am Soc Nephrol. (2017) 12:2024–31. doi: 10.2215/CJN.06100617, PMID: 29074817 PMC5718274

[3] Gibbons A Bayfield J Cinnirella M Draper H Johnson RJ Oniscu GC . Changes in quality of life (QoL) and other patient-reported outcome measures (PROMs) in living-donor and deceased-donor kidney transplant recipients and those awaiting transplantation in the UK ATTOM programme: a longitudinal cohort questionnaire survey with additional qualitative interviews. BMJ Open. (2021) 11:e047263. doi: 10.1136/bmjopen-2020-047263, PMID: 33853805 PMC8098938

[4] Schoot TS Goto NA van Marum RJ Hilbrands LB Kerckhoffs APM . Dialysis or kidney transplantation in older adults? A systematic review summarizing functional, psychological, and quality of life-related outcomes after start of kidney replacement therapy. Int Urol Nephrol. (2022) 54:2891–900. doi: 10.1007/s11255-022-03208-2, PMID: 35513758 PMC9534800

[5] Wong G Howard K Chapman JR Chadban S Cross N Tong A . Comparative survival and economic benefits of deceased donor kidney transplantation and dialysis in people with varying ages and co-morbidities. PloS One. (2012) 7:e29591. doi: 10.1371/journal.pone.0029591, PMID: 22279541 PMC3261160

[6] Zhang R . Donor-specific antibodies in kidney transplant recipients. Clin J Am Soc Nephrol. (2018) 13:182–92. doi: 10.2215/CJN.00700117, PMID: 28446536 PMC5753302

[7] Hiramitsu T Tomosugi T Futamura K Okada M Nishihira M Goto N . Optimal blood levels of (extended-release) tacrolimus in living donor kidney transplantation to prevent *de novo* donor-specific antibody production: a retrospective cohort study. Int Immunopharmacol. (2021) 91:107038. doi: 10.1016/j.intimp.2020.107038, PMID: 33388731

[8] Wiebe C Rush DN Nevins TE Birk PE Blydt-Hansen T Gibson IW . Class II eplet mismatch modulates tacrolimus trough levels required to prevent donor-specific antibody development. J Am Soc Nephrol. (2017) 28:3353–62. doi: 10.1681/ASN.2017030287, PMID: 28729289 PMC5661295

[9] Samaniego M Samaniego MC Barrio MC Potena L Zeevi A Djamali A . The influence of immunosuppressive agents on the risk of *de novo* donor-specific HLA antibody production in solid organ transplant recipients. Transplantation. (2016) 100:39–53. doi: 10.1097/TP.0000000000000869, PMID: 26680372 PMC4683034

[10] Benoni H Eloranta S Dahle DO Svensson MHS Nordin A Carstens J . Relative and absolute cancer risks among Nordic kidney transplant recipients-a population-based study. Transpl Int. (2020) 33:1700–10. doi: 10.1111/tri.13734, PMID: 32896035 PMC7756726

[11] Vajdic CM McDonald SP McCredie MRE van Leeuwen MT Stewart JH Law M . Cancer incidence before and after kidney transplantation. JAMA. (2006) 296:2823–31. doi: 10.1001/jama.296.23.2823, PMID: 17179459

[12] Al-Adra D Al-Qaoud T Fowler K Wong G . *De novo* Malignancies after kidney transplantation. Clin J Am Soc Nephrol. (2022) 17:434–43. doi: 10.2215/CJN.14570920, PMID: 33782034 PMC8975024

[13] Dantal J Hourmant M Cantarovich D Giral M Blancho G Dreno B . Effect of long-term immunosuppression in kidney-graft recipients on cancer incidence: randomised comparison of two cyclosporin regimens. Lancet. (1998) 351:623–8. doi: 10.1016/S0140-6736(97)08496-1, PMID: 9500317

[14] Merzkani MA Bentall AJ Smith BH Benavides Lopez XB D’Costa MR Park WD . Death with function and graft failure after kidney transplantation: risk factors at baseline suggest new approaches to management. Transplant Direct. (2022) 8:e1273. doi: 10.1097/TXD.0000000000001273, PMID: 35047660 PMC8759617

[15] Rosales BM de la Mata N Vajdic CM Kelly PJ Wyburn K Webster AC . Cancer mortality in kidney transplant recipients: an Australian and New Zealand population-based cohort study, 1980–2013. Int J Cancer. (2020) 146:2703–11. doi: 10.1002/ijc.32585, PMID: 31340063

[16] Au EH Chapman JR Craig JC Lim WH Teixeira-Pinto A Ullah S . Overall and site-specific cancer mortality in patients on dialysis and after kidney transplant. J Am Soc Nephrol. (2019) 30:471–80. doi: 10.1681/ASN.2018090906, PMID: 30765426 PMC6405152

[17] Arend SM Mallat MJ Westendorp RJ van der Woude FJ van Es LA . Patient survival after renal transplantation; more than 25 years follow-up. Nephrol Dial Transplant. (1997) 12:1672–9. doi: 10.1093/ndt/12.8.1672, PMID: 9269647

[18] Au E Wong G Chapman JR . Cancer in kidney transplant recipients. Nat Rev Nephrol. (2018) 14:508–20. doi: 10.1038/s41581-018-0022-6, PMID: 29802400

[19] Farrugia D Mahboob S Cheshire J Begaj I Khosla S Ray D . Malignancy-related mortality following kidney transplantation is common. Kidney Int. (2014) 85:1395–403. doi: 10.1038/ki.2013.458, PMID: 24257690

[20] van de Wetering J Roodnat JI Hemke AC Hoitsma AJ Weimar W . Patient survival after the diagnosis of cancer in renal transplant recipients: a nested case-control study. Transplantation. (2010) 90:1542–6. doi: 10.1097/TP.0b013e3181ff1458, PMID: 21076383

[21] Fröhlich FA Halleck F Lehner L Schrezenmeier EV Naik M Schmidt D . *De-novo* Malignancies after kidney transplantation: A long-term observational study. PloS One. (2020) 15:e0242805. doi: 10.1371/journal.pone.0242805, PMID: 33253202 PMC7703884

[22] Lim WH Badve SV Wong G . Long-term allograft and patient outcomes of kidney transplant recipients with and without incident cancer – a population cohort study. Oncotarget. (2017) 8:77771–82. doi: 10.18632/oncotarget.20781, PMID: 29100424 PMC5652814

[23] Blosser CD Haber G Engels EA . Changes in cancer incidence and outcomes among kidney transplant recipients in the United States over a thirty-year period. Kidney Int. (2021) 99:1430–8. doi: 10.1016/j.kint.2020.10.018, PMID: 33159960 PMC8096865

[24] Au EHK Chapman JR Teixeira-Pinto A Craig JC Wong G . Variations in risk of cancer and death from cancer according to kidney allograft function, graft loss, and return to dialysis. Transplantation. (2023) 107:1359–64. doi: 10.1097/TP.0000000000004493, PMID: 36683232

[25] Attieh RM Wadei HM Mao MA Mao SA Pungpapong S Taner CB . The impact of induction therapy on the risk of posttransplant lymphoproliferative disorder in adult kidney transplant recipients with donor-recipient serological Epstein-Barr virus mismatch. Am J Transplant. (2024) 24:1486–94. doi: 10.1016/j.ajt.2024.02.028, PMID: 38447887

[26] Asderakis A Sabah TK Watkins WJ Khalid U Szabo L Stephens MR . Thymoglobulin versus alemtuzumab versus basiliximab kidney transplantation from donors after circulatory death. Kidney Int Rep. (2022) 7:732–40. doi: 10.1016/j.ekir.2022.01.1042, PMID: 35497810 PMC9039467

[27] Haller MC Royuela A Nagler EV Pascual J Webster AC . Steroid avoidance or withdrawal for kidney transplant recipients. Cochrane Database Syst Rev. (2016) 2016:CD005632. doi: 10.1002/14651858.CD005632.pub3, PMID: 27546100 PMC8520739

[28] Sapir-Pichhadze R Laprise C Beauchamp ME Kaouache M Zhang X Della Vecchia AD . Immunosuppression and cancer risk in kidney transplant recipients: a retrospective cohort study. Int J Cancer. (2024) 154:2043–53. doi: 10.1002/ijc.34875, PMID: 38345158

[29] Opelz G Döhler B . Lymphomas after solid organ transplantation: a collaborative transplant study report. Am J Transplant. (2004) 4:222–30. doi: 10.1046/j.1600-6143.2003.00325.x, PMID: 14974943

[30] Sampaio MS Cho YW Shah T Bunnapradist S Hutchinson IV . Association of immunosuppressive maintenance regimens with posttransplant lymphoproliferative disorder in kidney transplant recipients. Transplantation. (2012) 93:73–81. doi: 10.1097/TP.0b013e31823ae7db, PMID: 22129761

[31] Robson R Cecka JM Opelz G Budde M Sacks S . Prospective registry-based observational cohort study of the long-term risk of Malignancies in renal transplant patients treated with mycophenolate mofetil. Am J Transplant. (2005) 5:2954–60. doi: 10.1111/j.1600-6143.2005.01125.x, PMID: 16303010

[32] Hiramitsu T Shimamoto Y Himeno T Hasegawa Y Futamura K Okada M . Risk factors for cancer and the importance of screening in adult recipients of living-donor kidney transplant. Front Immunol. (2026) 16:1678309. doi: 10.3389/fimmu.2025.1678309, PMID: 41583478 PMC12827106

[33] Okamoto M Wakabayashi Y Higuchi A Kadotani Y Ogino S Ushigome H . Therapeutic drug monitoring of mycophenolic acid in renal transplant recipients. Transplant Proc. (2005) 37:859–60. doi: 10.1016/j.transproceed.2004.12.238, PMID: 15848556

[34] Metz DK Holford N Kausman JY Walker A Cranswick N Staatz CE . Optimizing mycophenolic acid exposure in kidney transplant recipients: time for target concentration intervention. Transplantation. (2019) 103:2012–30. doi: 10.1097/TP.0000000000002762, PMID: 31584924 PMC6756255

[35] Kreis H Oberbauer R Campistol JM Mathew T Daloze P Schena FP . Long-term benefits with sirolimus-based therapy after early cyclosporine withdrawal. J Am Soc Nephrol. (2004) 15:809–17. doi: 10.1097/01.asn.0000113248.59077.76, PMID: 14978184

[36] Alberú J Pascoe MD Campistol JM Schena FP Rial MDC Polinsky M . Lower Malignancy rates in renal allograft recipients converted to sirolimus-based, calcineurin inhibitor-free immunotherapy: 24-month results from the CONVERT trial. Transplantation. (2011) 92:303–10. doi: 10.1097/TP.0b013e3182247ae2, PMID: 21792049

[37] Lim WH Russ GR Wong G Pilmore H Kanellis J Chadban SJ . The risk of cancer in kidney transplant recipients may be reduced in those maintained on everolimus and reduced cyclosporine. Kidney Int. (2017) 91:954–63. doi: 10.1016/j.kint.2016.11.008, PMID: 28109543

[38] Opelz G Unterrainer C Süsal C Döhler B . Immunosuppression with mammalian target of rapamycin inhibitor and incidence of post-transplant cancer in kidney transplant recipients. Nephrol Dial Transplant. (2016) 31:1360–7. doi: 10.1093/ndt/gfw088, PMID: 27190384

[39] Knoll GA Kokolo MB Mallick R Beck A Buenaventura CD Ducharme R . Effect of sirolimus on Malignancy and survival after kidney transplantation: systematic review and meta-analysis of individual patient data. BMJ. (2014) 349:g6679. doi: 10.1136/bmj.g6679, PMID: 25422259 PMC4241732

[40] Kidney Disease: Improving Global Outcomes (KDIGO) Transplant Work Group . KDIGO clinical practice guideline for the care of kidney transplant recipients. Am J Transplant. (2009) 9:S1–155. doi: 10.1111/j.1600-6143.2009.02834.x, PMID: 19845597

[41] Yu TM Lin CC Shu KH Chuang YW Huang ST Chen CH . Increased risk of hepatic complications in kidney transplantation with chronic virus hepatitis infection: a nationwide population-based cohort study. Sci Rep. (2016) 6:21312. doi: 10.1038/srep21312, PMID: 26892933 PMC4759529

[42] Shirai H Suzuki M Tomita Y Iwadoh K Kai K Sannomiya A . Renal transplantation in patients with human T-cell lymphotropic virus type 1. Transplant Proc. (2012) 44:83–6. doi: 10.1016/j.transproceed.2011.11.021, PMID: 22310586

[43] Goto N Uchida K Tomosugi T Futamura K Okada M Hiramitsu T . Long-term prognosis in kidney transplant recipients with human T-cell leukemia virus type 1 infection. Transpl Infect Dis. (2020) 22:e13314. doi: 10.1111/tid.13314, PMID: 32386274 PMC9285410

[44] Saowapa S Polpichai N Siladech P Wannaphut C Tanariyakul M Wattanachayakul P . Evaluating Kaposi sarcoma in kidney transplant patients: a systematic review and meta-analysis. Cureus. (2024) 16:e52527. doi: 10.7759/cureus.52527, PMID: 38371002 PMC10874301

[45] Tajima T Martinez OM Bernstein D Boyd SD Gratzinger D Lum G . Epstein-Barr virus-associated post-transplant lymphoproliferative disorders in pediatric transplantation: a prospective multicenter study in the United States. Pediatr Transplant. (2024) 28:e14763. doi: 10.1111/petr.14763, PMID: 38682750 PMC11115376

[46] Allen UD Preiksaitis JK AST . Infectious Diseases Community of Practice. Post-transplant lymphoproliferative disorders, Epstein-Barr virus infection, and disease in solid organ transplantation: guidelines from the American Society of Transplantation Infectious Diseases Community of Practice. Clin Transplant. (2019) 33:e13652. doi: 10.1111/ctr.13652, PMID: 31230381

[47] Nailescu C Shew ML . Human papillomavirus infection-related cancer risk for kidney transplant recipients during adult life can be reduced by vaccination during childhood and adolescence. Front Pediatr. (2022) 10:1057454. doi: 10.3389/fped.2022.1057454, PMID: 36533243 PMC9749905

[48] Ma BM Yap DYH Yip TPS Hung IFN Tang SCW Chan TM . Vaccination in patients with chronic kidney disease-review of current recommendations and recent advances. Nephrol (Carlton). (2021) 26:5–11. doi: 10.1111/nep.13741, PMID: 32524684

[49] Penn I . Evaluation of the candidate with a previous Malignancy. Liver Transpl Surg. (1996) 2:109–13. 9346710

[50] Acuna SA Sutradhar R Kim SJ Baxter NN . Solid organ transplantation in patients with preexisting Malignancies in remission: a propensity score matched cohort study. Transplantation. (2018) 102:1156–64. doi: 10.1097/TP.0000000000002178, PMID: 29557910 PMC7228636

[51] Brattström C Granath F Edgren G Smedby KE Wilczek HE . Overall and cause-specific mortality in transplant recipients with a pretransplantation cancer history. Transplantation. (2013) 96:297–305. doi: 10.1097/TP.0b013e31829854b7, PMID: 23759880

[52] Livingston-Rosanoff D Foley DP Leverson G Wilke LG . Impact of pre-transplant Malignancy on outcomes after kidney transplantation: United Network for Organ Sharing Database Analysis. J Am Coll Surg. (2019) 229:568–79. doi: 10.1016/j.jamcollsurg.2019.06.001, PMID: 31666186 PMC6879822

[53] Zwald F Leitenberger J Zeitouni N Soon S Brewer J Arron S . Recommendations for solid organ transplantation for transplant candidates with a pretransplant diagnosis of cutaneous squamous cell carcinoma, Merkel cell carcinoma and melanoma: a consensus opinion from the International Transplant Skin Cancer Collaborative (ITSCC). Am J Transplant. (2016) 16:407–13. doi: 10.1111/ajt.13593, PMID: 26820755

[54] Al-Adra DP Hammel L Roberts J Woodle ES Levine D Mandelbrot D . Pretransplant solid organ Malignancy and organ transplant candidacy: a consensus expert opinion statement. Am J Transplant. (2021) 21:460–74. doi: 10.1111/ajt.16318, PMID: 32969590 PMC8576374

[55] Al-Adra DP Hammel L Roberts J Woodle ES Levine D Mandelbrot D . Preexisting melanoma and hematological Malignancies, prognosis, and timing to solid organ transplantation: a consensus expert opinion statement. Am J Transplant. (2021) 21:475–83. doi: 10.1111/ajt.16324, PMID: 32976703 PMC8555431

[56] Bigotte Vieira M Arai H Nicolau C Murakami N . Cancer screening and cancer treatment in kidney transplant recipients. Kidney360. (2024) 5:1569–83. doi: 10.34067/KID.0000000000000545, PMID: 39480669 PMC11556922

[57] Hiramitsu T Himeno T Shimamoto Y Hasegawa Y Futamura K Okada M . Cancer after kidney transplantation. J Jpn Soc Clin Ren Transpl. (2025) 13:100–7. doi: 10.69408/jscrt.13.2_100, PMID: 41862425

[58] Chadban SJ Ahn C Axelrod DA Foster BJ Kasiske BL Kher V . KDIGO clinical practice guideline on the evaluation and management of candidates for kidney transplantation. Transplantation. (2020) 104:S11–103. doi: 10.1097/TP.0000000000003136, PMID: 32301874

[59] NHS Blood and Transplant . Annual activity report. Section 5 Kidney Activity (2025). Available online at: https://nhsbtdbe.blob.core.windows.net/umbraco-assets-corp/36811/section-5-kidney-activity.pdf (Accessed December 29, 2025).

[60] Berger JC Muzaale AD James N Hoque M Wang JMG Montgomery RA . Living kidney donors ages 70 and older: recipient and donor outcomes. Clin J Am Soc Nephrol. (2011) 6:2887–93. doi: 10.2215/CJN.04160511, PMID: 22034505 PMC3255359

[61] Desai R Collett D Watson CJ Johnson P Evans T Neuberger J . Cancer transmission from organ donors-unavoidable but low risk. Transplantation. (2012) 94:1200–7. doi: 10.1097/TP.0b013e318272df41, PMID: 23269448

[62] Eccher A Lombardini L Girolami I Puoti F Zaza G Gambaro G . How safe are organs from deceased donors with neoplasia? The results of the Italian Transplantation Network. J Nephrol. (2019) 32:323–30. doi: 10.1007/s40620-018-00573-z, PMID: 30604151

[63] SaBTO . Transplantation of organs from deceased donors with cancer or a history of cancer. London: Department of Health and Social Care. Advisory Committee on the Safety of Blood, Tissues and Organs (2020). Available online at: https://assets.publishing.service.gov.uk/media/5fe49ac68fa8f56af97b1e6d/transplantation_of_organs_from_deceased_donors_with_cancer_or_a_history_of_cancer-revised_FINAL44266_JNcw_cw.pdf (Accessed March 19, 2026).

[64] European Directorate for the Quality of Medicines & Health . Care (EDQM), Council of Europe. Guide to the quality and safety of organs for transplantation. 8th ed. Strasbourg: Council of Europe (2022). Available online at: https://www.edqm.eu/en/guide-quality-and-safety-of-organs-for-transplantation (Accessed March 19, 2026).

[65] The Transplantation Society of Australia And New Zealand (TSANZ) . TSANZ clinical guidelines for organ transplantation. Sydney: TSANZ (2023). Available online at: https://tsanz.com.au/storage/documents/TSANZ_Clinical_Guidelines_Version-111_13062023Final-Version.pdf (Accessed March 19, 2026).

[66] Lentine KL Kasiske BL Levey AS Adams PL Alberú J Bakr MA . KDIGO clinical practice guideline on the evaluation and care of living kidney donors. Transplantation. (2017) 101:S1–109. doi: 10.1097/TP.0000000000001769, PMID: 28742762 PMC5540357

[67] Liu-Smith F Jia J Zheng Y . UV-induced molecular signaling differences in melanoma and non-melanoma skin cancer. Adv Exp Med Biol. (2017) 996:27–40. doi: 10.1007/978-3-319-56017-5_3, PMID: 29124688

[68] Mittal A Colegio OR . Skin cancers in organ transplant recipients. Am J Transplant. (2017) 17:2509–530. doi: 10.1111/ajt.14382, PMID: 28556451

[69] Huo Z Li C Xu X Ge F Wang R Wen Y . Cancer risks in solid organ transplant recipients: results from a comprehensive analysis of 72 cohort studies. Oncoimmunology. (2020) 9:1848068. doi: 10.1080/2162402X.2020.1848068, PMID: 33299661 PMC7714465

[70] Fuente MJ Sabat M Roca J Lauzurica R Fernández-Figueras MT Ferrándiz C . A prospective study of the incidence of skin cancer and its risk factors in a Spanish Mediterranean population of kidney transplant recipients. Br J Dermatol. (2003) 149:1221–6. doi: 10.1111/j.1365-2133.2003.05740.x, PMID: 14674900

[71] Thet Z Lam AKY Ng SK Aung SY Han T Ranganathan D . An integrated skin cancer education program in renal transplant recipients and patients with glomerular disease. BMC Nephrol. (2022) 23:361. doi: 10.1186/s12882-022-02997-z, PMID: 36357857 PMC9647923

[72] Schiffman JD Fisher PG Gibbs P . Early detection of cancer: past, present, and future. Am Soc Clin Oncol Educ Book. (2015), 57–65. doi: 10.14694/EdBook_AM.2015.35.57, PMID: 25993143

[73] Wong G Chapman JR Craig JC . Cancer screening in renal transplant recipients: what is the evidence? Clin J Am Soc Nephrol. (2008) 3:S87–100. doi: 10.2215/CJN.03320807, PMID: 18309007 PMC3152279

[74] Acuna SA Huang JW Scott AL Micic S Daly C Brezden-Masley C . Cancer screening recommendations for solid organ transplant recipients: a systematic review of clinical practice guidelines. Am J Transplant. (2017) 17:103–14. doi: 10.1111/ajt.13978, PMID: 27575845

[75] Buxeda A Redondo-Pachón D Pérez-Sáez MJ Crespo M Pascual J . Sex differences in cancer risk and outcomes after kidney transplantation. Transplant Rev (Orlando). (2021) 35:100625. doi: 10.1016/j.trre.2021.100625, PMID: 34020178

[76] Kasiske BL Vazquez MA Harmon WE Brown RS Danovitch GM Gaston RS . Recommendations for the outpatient surveillance of renal transplant recipients. American Society of Transplantation. J Am Soc Nephrol. (2000) 11:S1–86. doi: 10.1681/ASN.V11suppl_1s1 11044969

[77] Knoll GA Blydt-Hansen TD Campbell P Cantarovich M Cole E Fairhead T . Canadian Society of Transplantation and Canadian Society of Nephrology commentary on the 2009 KDIGO clinical practice guideline for the care of kidney transplant recipients. Am J Kidney Dis. (2010) 56:219–46. doi: 10.1053/j.ajkd.2010.05.004, PMID: 20659623

[78] Chadban SJ Barraclough KA Campbell SB Clark CJ Coates PT Cohney SJ . KHA-CARI guideline: KHA-CARI adaptation of the KDIGO clinical practice guideline for the care of kidney transplant recipients. Nephrol (Carlton). (2012) 17:204–14. doi: 10.1111/j.1440-1797.2011.01559.x, PMID: 22212251

[79] EBPG Expert Group on Renal Transplantation . European best practice guidelines for renal transplantation. Section IV: Long-term management of the transplant recipient. IV.6.3. Cancer risk after renal transplantation. Solid organ cancers: prevention and treatment. Nephrol Dial Transplant. (2002) 17:32–6. doi: 10.1093/ndt/17.suppl_4.3 12091641

[80] Baker R Jardine A Andrews P . Renal Association Clinical Practice Guideline on post-operative care of the kidney transplant recipient. Nephron Clin Pract. (2011) 118:c311–47. doi: 10.1159/000328074, PMID: 21555902

[81] Schrem H Schneider V Kurok M Goldis A Dreier M Kaltenborn A . Independent pre-transplant recipient cancer risk factors after kidney transplantation and the utility of G-chart analysis for clinical process control. PloS One. (2016) 11:e0158732. doi: 10.1371/journal.pone.0158732, PMID: 27398803 PMC4939933

[82] Massicotte-Azarniouch D Detwiler RK Hu Y Falk RJ Saha MK Hogan SL . Malignancy risk in kidney transplant recipients exposed to immunosuppression pre-transplant for the treatment of glomerulonephritis. Nephrol Dial Transplant. (2023) 38:2009–18. doi: 10.1093/ndt/gfac337, PMID: 36549661 PMC10468752

[83] Bamoulid J Courivaud C Coaquette A Crépin T Carron C Gaiffe E . Late persistent positive EBV viral load and risk of solid cancer in kidney transplant patients. Transplantation. (2017) 101:1473–8. doi: 10.1097/TP.0000000000001280, PMID: 27367471

[84] Imamura R Nakazawa S Yamanaka K Kakuta Y Tsutahara K Taniguchi A . Cumulative cancer incidence and mortality after kidney transplantation in Japan: a long-term multicenter cohort study. Cancer Med. (2021) 10:2205–15. doi: 10.1002/cam4.3636, PMID: 33314709 PMC7982608

[85] Bretagnol A Halimi JM Roland M Barbet C Machet L Al Najjar AA . Autosomal dominant polycystic kidney disease: risk factor for nonmelanoma skin cancer following kidney transplantation. Transpl Int. (2010) 23:878–86. doi: 10.1111/j.1432-2277.2010.01070.x, PMID: 20230542

[86] Skov Dalgaard L Fassel U Østergaard LJ Jespersen B Schmeltz Søgaard OS Jensen-Fangel S . Risk of human papillomavirus-related cancers among kidney transplant recipients and patients receiving chronic dialysis – an observational cohort study. BMC Nephrol. (2013) 14:137. doi: 10.1186/1471-2369-14-137, PMID: 23834996 PMC3710213

[87] Gupta G Kuppachi S Kalil RS Buck CB Lynch CF Engels EA . Treatment for presumed BK polyomavirus nephropathy and risk of urinary tract cancers among kidney transplant recipients in the United States. Am J Transplant. (2018) 18:245–52. doi: 10.1111/ajt.14530, PMID: 28980390 PMC5739985

[88] Viecelli AK Lim WH Macaskill P Chapman JR Craig JC Clayton P . Cancer-specific and all-cause mortality in kidney transplant recipients with and without previous cancer. Transplantation. (2015) 99:2586–92. doi: 10.1097/TP.0000000000000760, PMID: 26102612

[89] Lv GJ Ji D Yu L Chen HY Chen J He M . Risk of hepatocellular carcinoma occurrence after antiviral therapy for patients with chronic hepatitis C Infection: a systematic review and meta-analysis. Hepatol Int. (2024) 18:1459–71. doi: 10.1007/s12072-024-10700-7, PMID: 38965190

[90] Tahata Y Sakamori R Yamada R Kodama T Hikita H Nozaki Y . Risk of hepatocellular carcinoma after sustained virologic response in hepatitis C virus patients without advanced liver fibrosis. Hepatol Res. (2022) 52:824–32. doi: 10.1111/hepr.13806, PMID: 35749289

[91] Murai K Hikita H Yamada R Nishimura Y Miyazaki M Ishida H . The risk of developing hepatocellular carcinoma persists in chronic hepatitis B patients even after the long-term administration of nucleos(t)ide analogs. Hepatol Res. (2025) 55:1228–38. doi: 10.1111/hepr.14225, PMID: 40522439

[92] Cai S Tan X Miao K Li D Cheng S Li P . Effectiveness and safety of therapeutic vaccines for precancerous cervical lesions: a systematic review and meta-analysis. Front Oncol. (2022) 12:918331. doi: 10.3389/fonc.2022.918331, PMID: 35734598 PMC9207463

[93] Nashan B Citterio F . Wound healing complications and the use of mammalian target of rapamycin inhibitors in kidney transplantation: a critical review of the literature. Transplantation. (2012) 94:547–61. doi: 10.1097/TP.0b013e3182551021, PMID: 22941182

[94] Mabood Khalil MA Al-Ghamdi SMG Dawood US Ahmed Khamis SS Ishida H Chong VH . Mammalian target of rapamycin inhibitors and wound healing complications in kidney transplantation: old myths and new realities. J Transplant. (2022) 2022:6255339. doi: 10.1155/2022/6255339, PMID: 35265364 PMC8901320

[95] Maggiore U Palmisano A Buti S Claire Giudice GC Cattaneo D Giuliani N . Chemotherapy, targeted therapy and immunotherapy: which drugs can be safely used in the solid organ transplant recipients? Transpl Int. (2021) 34:2442–58. doi: 10.1111/tri.14115, PMID: 34555228 PMC9298293

[96] Rosner MH Jhaveri KD McMahon BA Perazella MA . Onconephrology: the intersections between the kidney and cancer. CA Cancer J Clin. (2021) 71:47–77. doi: 10.3322/caac.21636, PMID: 32853404

[97] Porta C Cosmai L Gallieni M Pedrazzoli P Malberti F . Renal effects of targeted anticancer therapies. Nat Rev Nephrol. (2015) 11:354–70. doi: 10.1038/nrneph.2015.15, PMID: 25734768

[98] Perazella MA Moeckel GW . Nephrotoxicity from chemotherapeutic agents: clinical manifestations, pathobiology, and prevention/therapy. Semin Nephrol. (2010) 30:570–81. doi: 10.1016/j.semnephrol.2010.09.005, PMID: 21146122

[99] Perazella MA . Onco-nephrology: renal toxicities of chemotherapeutic agents. Clin J Am Soc Nephrol. (2012) 7:1713–21. doi: 10.2215/CJN.02780312, PMID: 22879440

[100] Hartmann EL Gatesman M Roskopf-Somerville J Stratta R Farney A Sundberg A . Management of leukopenia in kidney and pancreas transplant recipients. Clin Transplant. (2008) 22:822–8. doi: 10.1111/j.1399-0012.2008.00893.x, PMID: 19040562

[101] Yang Y Yu B Chen Y . Blood disorders typically associated with renal transplantation. Front Cell Dev Biol. (2015) 3:18. doi: 10.3389/fcell.2015.00018, PMID: 25853131 PMC4365751

[102] Rolston KVI . The Infectious Diseases Society of America 2002 guidelines for the use of antimicrobial agents in patients with cancer and neutropenia: salient features and comments. Clin Infect Dis. (2004) 39:S44–8. doi: 10.1086/383053, PMID: 15250020

[103] Smith TJ Bohlke K Lyman GH Carson KR Crawford J Cross SJ . Recommendations for the use of WBC growth factors: American Society of Clinical Oncology Clinical Practice Guideline Update. J Clin Oncol. (2015) 33:3199–212. doi: 10.1200/JCO.2015.62.3488, PMID: 26169616

[104] Murakami N Mulvaney P Danesh M Abudayyeh A Diab A Abdel-Wahab N . A multi-center study on safety and efficacy of immune checkpoint inhibitors in cancer patients with kidney transplant. Kidney Int. (2021) 100:196–205. doi: 10.1016/j.kint.2020.12.015, PMID: 33359528 PMC8222056

[105] d’Izarny-Gargas T Durrbach A Zaidan M . Efficacy and tolerance of immune checkpoint inhibitors in transplant patients with cancer: a systematic review. Am J Transplant. (2020) 20:2457–65. doi: 10.1111/ajt.15811, PMID: 32027461

[106] Bolufer M Soler J Molina M Taco O Vila A Macía M . Immunotherapy for cancer in kidney transplant patients: a difficult balance between risks and benefits. Transpl Int. (2024) 37:13204. doi: 10.3389/ti.2024.13204, PMID: 39654653 PMC11625584

[107] June CH Sadelain M . Chimeric antigen receptor therapy. N Engl J Med. (2018) 379:64–73. doi: 10.1056/NEJMra1706169, PMID: 29972754 PMC7433347

[108] Krishnamoorthy S Ghobadi A Santos RD Schilling JD Malone AF Murad H . CAR-T therapy in solid organ transplant recipients with treatment refractory posttransplant lymphoproliferative disorder. Am J Transplant. (2021) 21:809–14. doi: 10.1111/ajt.16367, PMID: 33089906

[109] McKenna M Epperla N Ghobadi A Liu J Lazaryan A Ibrahim U . Real-world evidence of the safety and survival with CD19 CAR-T cell therapy for relapsed/refractory solid organ transplant-related PTLD. Br J Haematol. (2023) 202:248–55. doi: 10.1111/bjh.18828, PMID: 37129856

[110] Hellemans R Pengel LHM Choquet S Maggiore U . for ESOT Workstream 3 of the TLJ (Transplant Learning Journey) project. Managing immunosuppressive therapy in potentially cured post-kidney transplant cancer (excluding non-melanoma skin cancer: an overview of the available evidence and guidance for shared decision-making. Transpl Int. (2021) 34:1789–800. doi: 10.1111/tri.13952, PMID: 34146426 PMC8518116

[111] Gatault P Kamar N Büchler M Colosio C Bertrand D Durrbach A . Reduction of extended-release tacrolimus dose in low-immunological-risk kidney transplant recipients increases risk of rejection and appearance of donor-specific antibodies: a randomized study. Am J Transplant. (2017) 17:1370–9. doi: 10.1111/ajt.14109, PMID: 27862923

[112] Dantal J Morelon E Rostaing L Goffin E Brocard A Tromme I . Sirolimus for secondary prevention of skin cancer in kidney transplant recipients: 5-year results. J Clin Oncol. (2018) 36:2612–20. doi: 10.1200/JCO.2017.76.6691, PMID: 30016177

